# The lunar nodal phase cycle and winter atmospheric pressure as possible determinants of moth abundance: Analyses of a 30‐year time series from South Norway

**DOI:** 10.1002/ece3.9443

**Published:** 2022-10-27

**Authors:** Vidar Selås, Sverre Kobro

**Affiliations:** ^1^ Department of Ecology and Natural Resource Management Norwegian University of Life Sciences Ås Norway; ^2^ Norwegian Institute of Bioeconomy Research Ås Norway

**Keywords:** air pressure, herbivores, Lepidoptera, Moon, population fluctuations

## Abstract

According to the plant stress hypothesis, population peaks of herbivores such as moths are caused by plant stress factors that force plants to reallocate stored defensive proteins to transportable and easily digestive N‐compounds. A suggested plant stress factor is ionization caused by cosmic ray muons, which are modulated by the 9.3‐year lunar nodal phase cycle, solar activity, and atmospheric pressure. Vascular plants are more sensitive to ionization than are bryophytes, and woody plants are more sensitive than are herbaceous plants, but the difference may be less during dormancy in winter. We selected the 14 most common moth species from a 30‐year light‐trapping study in southern Norway to test whether the fluctuation patterns of species from three different feeding guilds were correlated with lunar/solar cycles, or with atmospheric pressure in winter, when muon fluxes are higher than in other seasons. The population indices of three species feeding on deciduous woody plants were positively correlated with the lunar nodal phase index, and there was a similar tendency for the remaining three species. No positive correlations with the lunar index were found for species feeding on herbs or mosses. For nine species, that is, from all three guilds, there was a significant negative correlation between the population index and winter atmospheric pressure in the previous year. The results are in accordance with predictions deduced from the cosmic ray hypothesis, but thorough investigations of the proposed physiological mechanisms are needed for the hypothesis to be widely accepted.

## Graphical abstract

Among 14 moth species from a 30‐year light‐trapping study, the annual population indices of three of six species (feeding on deciduous woody plants) were significantly related to an index of the 9.3‐year lunar nodal phase cycle. For nine species, there was a negative relationship with the winter atmospheric pressure in the previous year. The causality could be that the position of the Moon relative to the Earth and the Sun, and atmospheric pressure, affects surface muon fluxes, a possible plant stress factor.

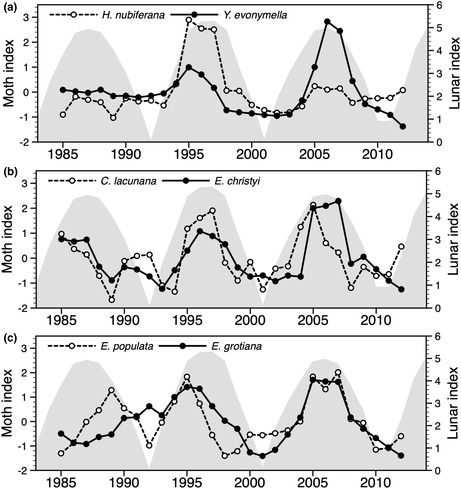

## 1 INTRODUCTION

Multiannual population fluctuations with a periodicity of approximately 10 years have been reported for several herbivore species. By use of dendrochronology, Esper et al. (2007) showed that the regular outbreaks of larch budmoth *Zeirophera diniana* in the European Alps have persisted over a period of 1173 years, with a mean cycle period of 9.3 years. No reason for this periodicity was suggested, but in a less‐known study, published 30 years earlier, Archibald (1977) linked the 9.3‐year population cycle of snowshoe hare *Lepus americanus* and ruffed grouse *Bonasa umbellus* in Canada to the 9.3‐year lunar nodal phase cycle (see also Archibald, 2014). A 9.3‐year signal is apparent also in a 120‐year time series of the autumnal moth *Epirrita autumnata* in Scandinavia (Selås, 2014). Common to these herbivores is that they feed on deciduous woody plants and that they live in areas with low protection against cosmic rays, that is, at high altitude or high geomagnetic latitude.

According to the plant stress hypothesis, herbivores benefit from plant stress factors that force plants to reallocate stored defense proteins to transportable and thus easily available nitrogen compounds (White, 1984). If changes in the ratio between protective and digestive proteins raise the protein digestibility per time unit above the herbivore's critical threshold, and this happens synchronously in a population of plants, there will be a strong temporal increase in the carrying capacity of the herbivore (White, 1993). One possible plant stress factor is ionizing radiation (De Micco et al., 2011; White, 1984).

It was not possible in the 1970s to suggest how the Moon could influence herbivore abundance, so the work of Archibald (1977) did not get much attention then. However, thanks to advances in astrophysics and atmospheric research, we now know that the position of the Moon affects the magnetic connection between the Sun and the Earth, and thereby the atmospheric protection against cosmic ray muons (Selås, 2014, and references therein). Muons are secondary cosmic ray particles, created by collisions between primary galactic cosmic rays (mainly protons) and air molecules, and they are the cosmic ray compounds that most strongly affect life on Earth (Ferrari & Szuszkiewicz, 2009). Because plants mobilize proteins to repair damages caused by ionizing radiation (De Micco et al., 2011), ionization caused by muons may be a plant stress factor that increases the protein digestibility for herbivores. Vascular plants are in general more sensitive to ionizing radiation than are bryophytes, and woody plants are more sensitive than are herbaceous plants (Caplin et al., 2020; Govindapyari et al., 2010).

The amount of galactic cosmic rays that reach the Earth is also reduced in periods with high solar activity. In addition, there is an inverse correlation between surface muon fluxes and atmospheric pressure, because cosmic ray particles need more energy to penetrate the atmosphere and reach the ground when the atmospheric pressure is high (De Mendonca et al., 2016). In the northern hemisphere, there is a peak in muon fluxes in winter (Acero et al., 2021; de Mendonca et al., 2016), when the mean atmospheric pressure varies more and reach lower levels than in spring and summer. Hence, winter atmospheric pressure is a factor that may affect plant performance and thus future herbivore numbers. However, to our knowledge, atmospheric pressure in previous winters has so far not been addressed in studies of herbivore population fluctuations.

Although several examples of the predicted relationships between plant stress and herbivore performance exist, the proposed physiological mechanisms need further investigations. To encourage such projects, which undoubtedly will be both extensive and expensive, any additional evidence for relationships between plant stress and herbivore numbers, in particular from long‐term studies, should be welcome. Note here that the plant stress factors in question are mainly those that require increased metabolic activity in the plants, and not those that may rather lead to inactivity, such as summer drought.

Here, we test whether patterns in a 30‐year light‐trapping data series of nocturnal moths from southern Norway are in accordance with predictions deduced from the cosmic ray hypothesis. By selecting the 14 most common species, we obtained species from three different guilds, feeding on deciduous woody plants (trees/bushes/dwarf shrubs), herbaceous plants, and mosses, respectively. Mosses also rely on protein‐based defense (Markham et al., 2006), so the plant stress hypothesis may apply here as well. Our prediction was that particularly population indices of moths feeding on deciduous woody plants, which are more sensitive to irradiation in the growing season than herbs and bryophytes (De Micco et al., 2011; Woodwell, 1962), should show a positive correlation with an index of the 9.3‐year lunar nodal phase cycle, or alternatively a negative correlation with an index of the 11‐year solar cycle (Selås, 2014). Because we expected less differences in plants' resistance to irradiation during the inactive winter period, we predicted that population indices of all moth species may be negatively related to indices of atmospheric pressure in the previous winter.

## 2 MATERIALS AND METHODS

### 2.1 Study area

The study area is situated in Nesodden municipality in Akershus County (59°44′N, 10°36′E, 70 m elevation), 20 km south of Oslo, the capital of Norway. The landscape consists of coniferous, deciduous, and mixed forests, open grassland, and gardens. For more detailed descriptions of the study area see Selås et al. (2013) and Burner et al. (2021).

### 2.2 Moth light trapping

During 1984–2013, one light trap was usually operated three nights each week from late June to mid or late October. Earliest trapping date was 23. July and latest 31. October. The annual number of trap nights varied from 45 to 57 (mean 51.4, SD = 3.65). The trap was a simple funnel type with a 160 W mixed light bulb (Osram HWL 160 W/235 V), situated with the bulb 1 m above the ground at the same location each year.

For the present analyses, we selected the most common species based on the trapping index described by Kobro (1991), that is, all species with index ≥3.0, assuming that results for the most common species would be most reliable. One of these species was abundant only the first 2 years of the study period and was therefore excluded. The remaining sample included 14 species, of which six feed on deciduous trees, bushes, or dwarf shrubs, four on herbs, and four on mosses (Table 1).

**TABLE 1:** Selected moth species (mean annual trapping index ≥3.0) from a 30‐year light‐trapping series from Nesodden, South Norway.

Species and family	Winter stage	Larvae food
1) Species feeding on deciduous woody plants		
*Yponomeuta evonymella* (45.8), Yponomeutidae	Egg	*Prunus padus*
*Epagoge grotiana* (44.3), Tortricidae	Egg	*Quercus*, *Rubus*, *Vaccinium*
*Hedya nubiferana* (103.3), Tortricidae	Larvae	*Sorbus*, *Crataegus*, *Prunus*
*Celypha lacunana* (71.7), Tortricidae	Egg	Deciduous and herbs
*Epirrita christyi* (52.5), Geometridae	Egg	Deciduous
*Eulithis populata* (73.1), Geometridae	Egg	*Vaccinium*
2) Species feeding on herbs		
*Plutella xylostella* (41.8), Plutellidae	Pupae	*Brassicaceae*
*Lathronympha strigana* (58.0), Tortricidae	Egg	*Hypericum* spp.
*Rusina ferruginea* (59.4), Noctuidae	Larvae	A wide range of herbs
*Noctua pronuba* (49.4), Noctuidae	Larvae	A wide range of herbs
3) Species feeding on mosses		
*Eudonia truncicolella* (621.8), Crambidae	Larvae	Mosses on the ground
*Eudonia lacustrata* (37.1), Crambidae	Pupae	Mosses on tree trunks
*Scoparia ambigualis* (40.1), Crambidae	Larvae	Mosses on the ground
*Catoptria falsella* (47.0), Crambidae	Larvae	Mosses on tree trunks

*Note*: For each species, the mean annual number trapped is given in parentheses.

### 2.3 Explanatory variables

**FIGURE 1:**
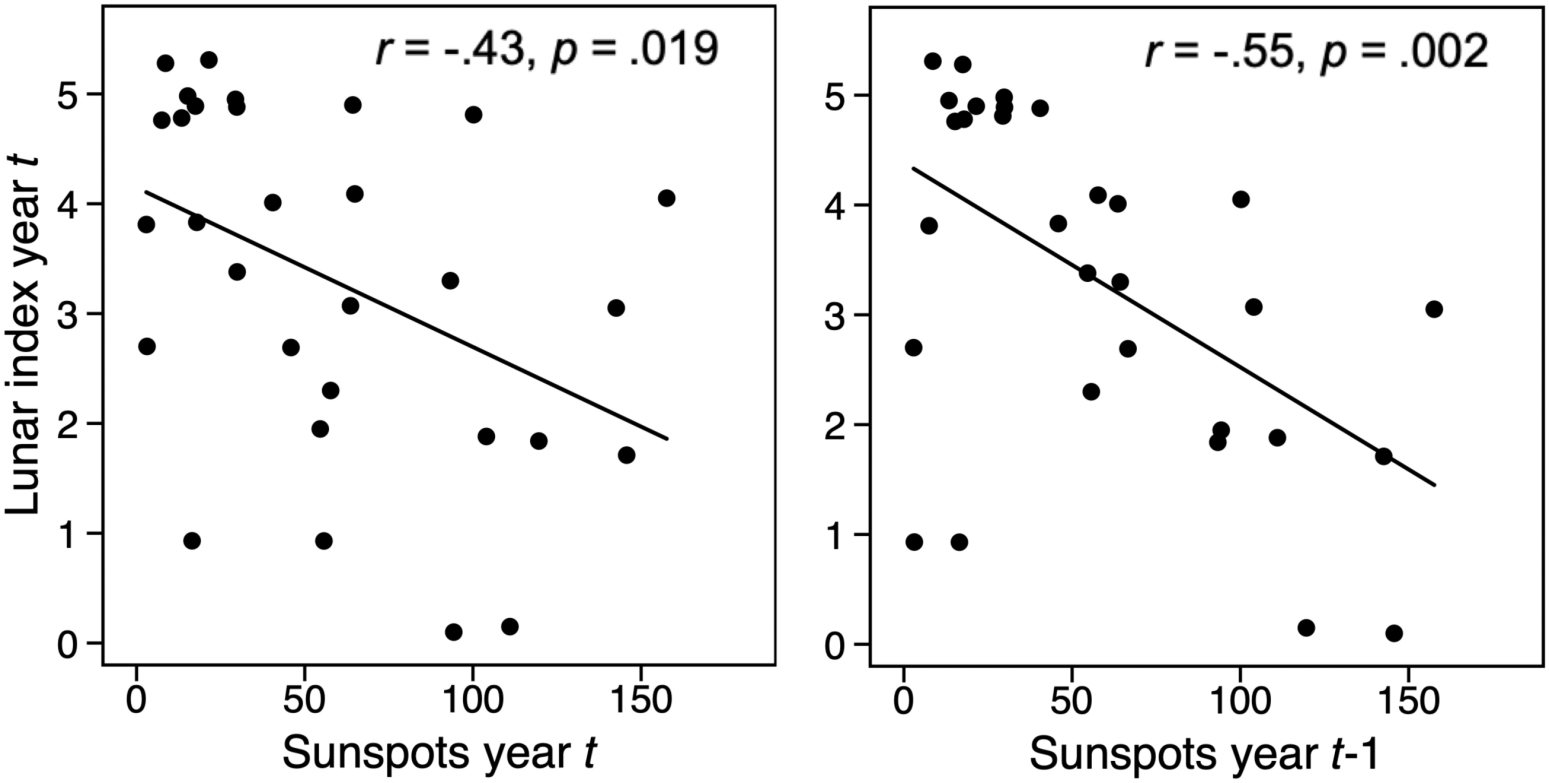
An index of the lunar nodal phase plotted against an index of solar activity (sunspots) in the current and previous year.

The lunar index was calculated as the absolute value of the difference between maximum lunar declination in the equinox month of September and the tilt angle between the ecliptic and the equatorial plane (Archibald, 2014; Selås, 2014). Surface muon fluxes will be positively related to this index (Selås, 2014). As a proxy for solar activity, we used the yearly mean of sunspot numbers. For the period 1984–2013, the lunar index was negatively correlated with the solar index (*r* = −.43, *p* = .019) and with the solar index of the previous year (*r* = −.55, *p* = .002; Figure 1).

When testing for relationships with winter atmospheric pressure, we used the mean monthly atmospheric pressure in each of the months: December, January, and February, giving three atmospheric indices. The reason for this was that we did not know whether high muon fluxes would affect plants already in early winter, or rather in late winter, when the light intensity is higher. Because the predicted protein mobilization in plants is expected to occur during the first summer, with recovery of the constitutive defense during the second, and because the impact of increased protein availability is assumed to be strongest on first instar larvae (White, 1993), a 1‐year time lag between winter atmospheric pressure and moth indices should be expected. We therefore used only winter atmospheric pressure of the previous year, and not of the winter prior to the moth trapping. By this approach, we also reduced the risk of confounding effects of possible direct effects of winter weather events related to atmospheric pressure.

The atmospheric data used are from Blindern Meteorological Station (data from the Norwegian Meteorological Institute; eKlima.no), situated 25 km from the trapping site. None of the atmospheric pressure indices used were significantly related to the lunar or solar indices (*p* > .1).

### 2.4 Statistical analyses

For each moth species, we tested for periodicity by use of spectral density analyses, and for time lags in relation to lunar/solar indices by use of cross‐correlation, with confidence intervals corrected for autocorrelations (Diggle, 1990). To reduce the impact of possible short‐term (2–4 year) fluctuations in these analyses, the moth series were transformed by calculating 3‐year running means. This transformation reduced the time series from 30 to 28 years. Thereafter we removed possible trends (see Burner et al., 2021) by using the residuals from a linear regression model with year as independent variable.

To investigate relationships between moth population indices and atmospheric pressure in December, January, and February in the previous year, we used annual changes (first differences) of the log‐transformed number of trapped individuals of each species, giving a time series of 29 years. Zero values were set to one in these analyses. This index gives the relative population change, positive or negative, from the previous year (year *t* − 1) to the current year (year *t*). In linear regression models for each species, there were no positive autocorrelations (which tend to give too low *p*‐values) in the residuals, and the residuals did not differ significantly from normal distribution.

Finally, we used the annual changes of the log‐transformed moth data as response variable in a linear mixed regression model with species as random effect and feeding guild (fixed variable, three levels), lunar index, atmospheric pressure, and the interaction between feeding guild and other predictors as explanatory variables. The interactions were included because the feeding guilds appeared to differ with regard to their relationship with both the lunar index and atmospheric pressure.

All tests were run with an alpha level of .05. The software used for all analyses was JMP®Pro 15.0.0 (SAS Institute).

## 3 RESULTS

**FIGURE 2:**
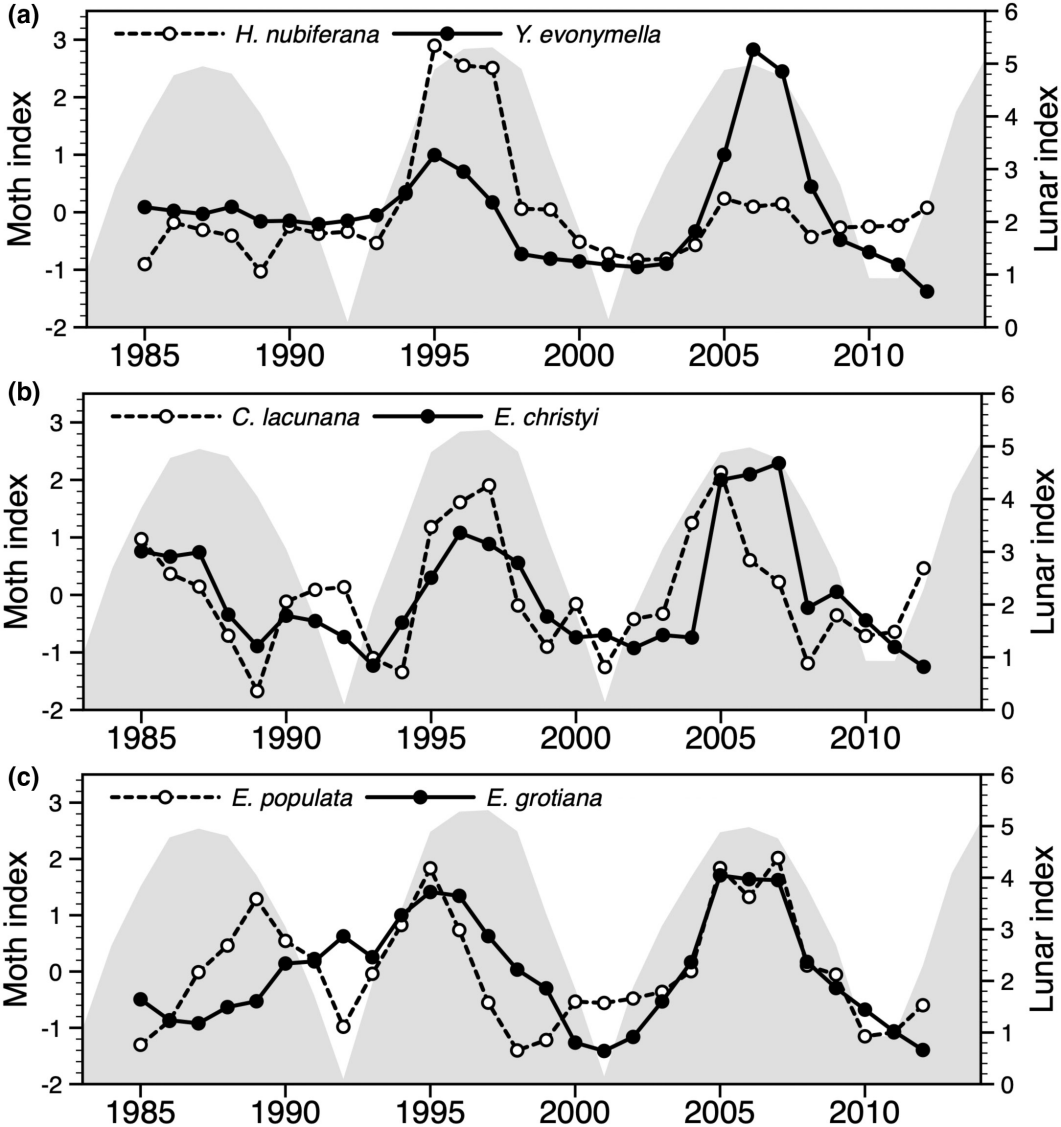
Standardized smoothed (3‐year running mean) and detrended population indices of six moth species feeding on deciduous woody plants, which were predicted to fluctuate in synchrony with the 9.3‐year lunar nodal phase cycle, given as shaded area in each panel. (a) Two species feeding mainly or to some extent on *Prunus*, (b) two species feeding on a wide variety of deciduous trees and bushes, (c) two species feeding mainly or to some extent on *Vaccinium*.

**FIGURE 3:**
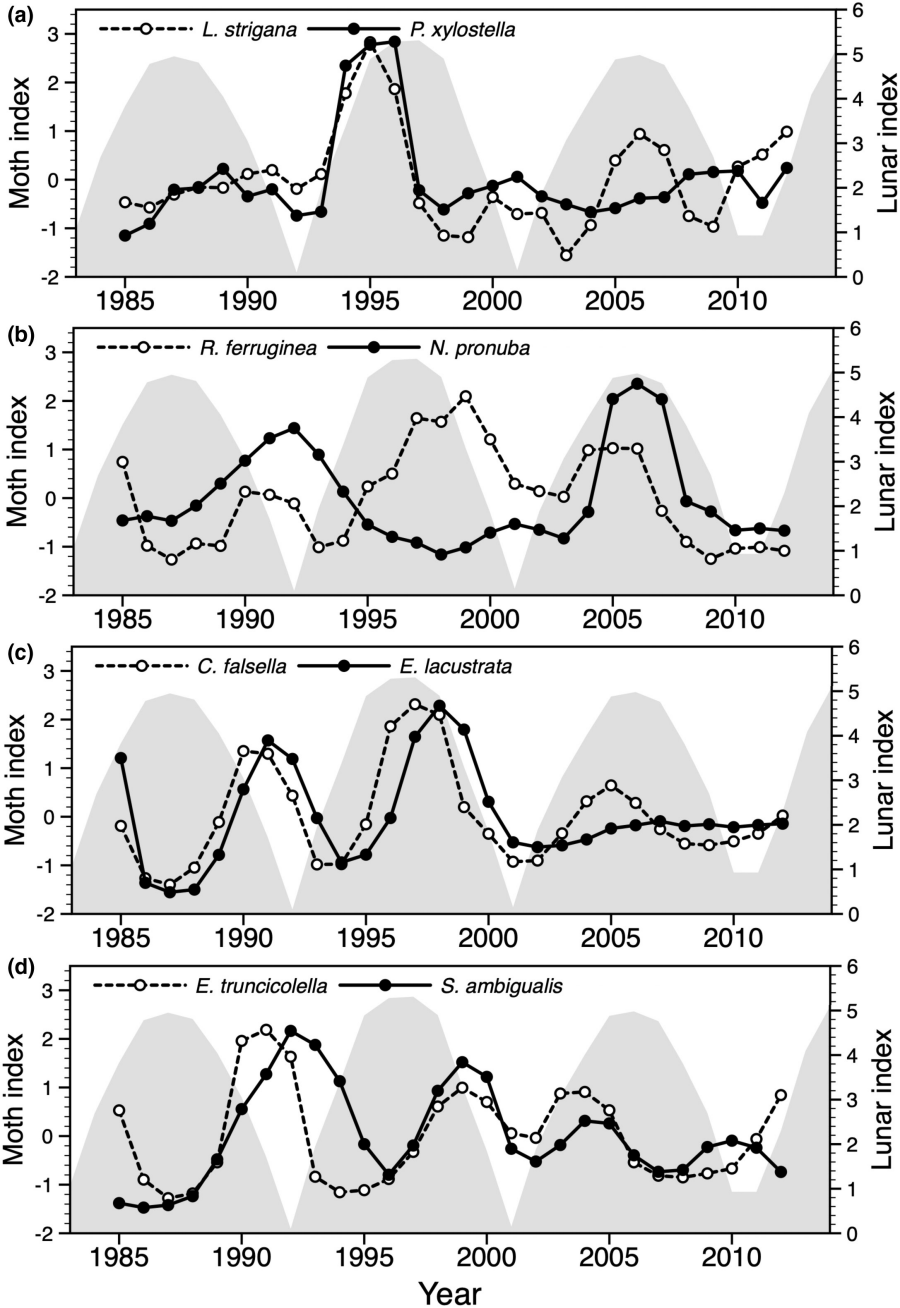
Standardized smoothed (3‐year running mean) and detrended population indices of eight moth species not predicted to fluctuate in synchrony with the 9.3‐year lunar nodal phase cycle, given as shaded area in each panel. (a) Two species feeding on a limited number of herbs, (b) two species feeding on a wide variety of herbs, (c) two species feeding on mosses growing on tree trunks and rocks, (d) two species feeding on mosses growing on the ground.

In analyses of smoothed (3‐year running mean) and detrended moth series, five of six species feeding on deciduous trees showed a significant periodicity (Table 2; Figure 2). For three of them, *Yponomeuta evonymella*, *Celypha lacunana*, and *Epirrita christyi*, the spectral density plot peaked at 9.3 years. This periodicity reflects the sample size (28) divided by number of peaks (3), and not necessarily the lunar nodal phase cycle. For the remaining species of this guild, the highest peak was at 14 years. Among species feeding on herbs, there was one with a periodicity of 5–6 years (*Lathronympha strigana*, five peaks), one with a periodicity of 7 years (*Rusina ferruginea*, four peaks), and one with a periodicity of 14 years (*Noctua pronuba*, two peaks). Three of four species feeding on mosses showed a significant periodicity of 7 years (Table 2; Figure 3).

**TABLE 2:** Results from spectral density analyses and cross‐correlation analyses with a 9.3‐year lunar index and the 11‐year solar index, for 14 moth species in a 30‐year light‐trapping series from South Norway.

Species	Spectral density	Cross‐correlation coefficients			
	Fisher's kappa	Lunar index		Solar index	
		0	1	0	1
1) Species feeding on deciduous woody plants					
*Yponomeuta evonymella*	5.29	0.57	0.46	−0.46	−0.32
	(**.025**)	(**.034**)	(.112)	(.121)	(.309)
*Epagoge grotiana*	7.63	0.48	0.34	−0.40	−0.18
	(**<.001**)	(.089)	(.259)	(.182)	(.565)
*Hedya nubiferana*	4.34	0.47	0.31	−0.42	−0.41
	(.098)	(.090)	(.283)	(.145)	(.168)
*Celypha lacunana*	5.01	0.49	0.26	−0.42	−0.25
	(**.038**)	(**.045**)	(.312)	(.100)	(.349)
*Epirrita christyi*	7.45	0.69	0.64	−0.56	−0.51
	(**<.001**)	(**.004**)	(**.011**)	(**.036**)	(.064)
*Eulithis populata*	5.56	0.48	0.30	−0.11	−0.07
	**(.016**)	(.071)	(.288)	(.698)	(.805)
2) Species feeding on herbs					
*Plutella xylostella*	3.36	0.20	0.06	−0.21	−0.20
	(.347)	(.478)	(.826)	(.465)	(.484)
*Lathronympha strigana*	4.87	0.17	−0.06	−0.25	−0.18
	(**.046**)	(.544)	(.839)	(.392)	(.558)
*Rusina ferruginea*	6.83	0.24	0.33	0.10	0.12
	(**.002**)	(.418)	(.272)	(.752)	(.698)
*Noctua pronuba*	8.57	0.04	0.07	0.02	0.22
	(**<.001**)	(.881)	(.819)	(.946)	(.492)
3) Species feeding on mosses					
*Eudonia truncicolella*	6.57	−0.41	−0.20	0.57	0.70
	(**.003**)	(.128)	(.495)	(**.030**)	(**.007**)
*Eudonia lacustrata*	6.25	−0.13	0.16	0.18	0.20
	(**.005**)	(.658)	(.574)	(.531)	(.492)
*Scoparia ambigualis*	4.42	−0.45	−0.28	0.39	0.52
	(.088)	(.112)	(.357)	(.193)	(.080)
*Catoptria falsella*	7.29	0.26	0.39	0.09	0.07
	(**<.001**)	(.366)	(.172)	(.761)	(.826)

*Note*: Only correlation coefficients without time lag, and with 1‐year lag, are given. *p*‐Values are given in brackets, with values <.05 in bold.

**FIGURE 4:**
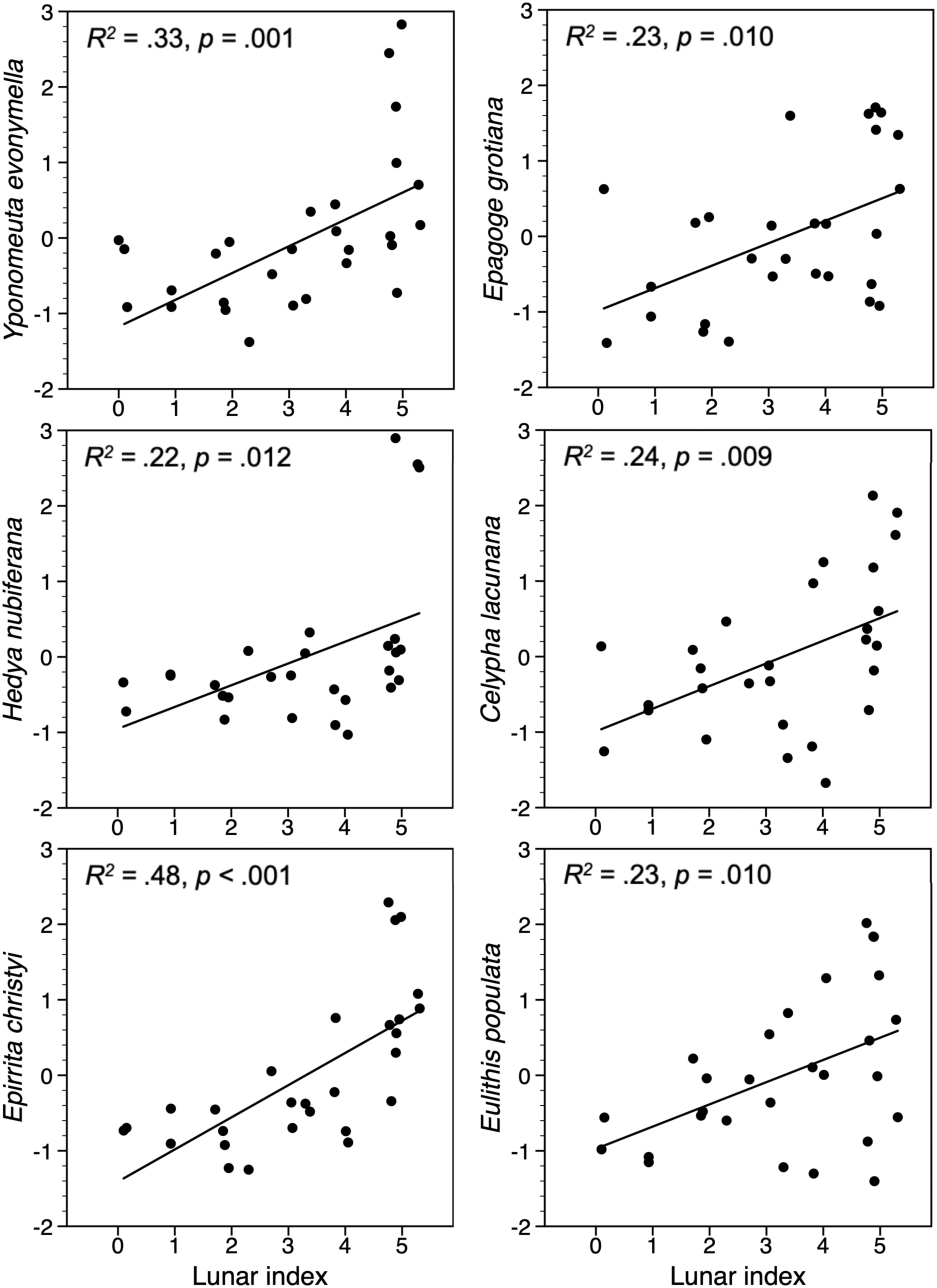
Standardized smoothed (3‐year running mean) and detrended population indices of six moth species feeding on deciduous woody plants, plotted against an index of the 9.3‐year lunar nodal phase cycle.

In cross‐correlation analyses, the smoothed and detrended indices of three species feeding on deciduous woody plants were significantly correlated with the lunar index, without time lag, and there was a similar tendency (*p* < .1) for the remaining three species of this guild (Table 2; Figure 2). When the 9.3‐year lunar index was substituted by the 11‐year solar index, the highest absolute value of the correlation coefficients was reduced for all six species (Table 2). In linear regression models, where autocorrelations in the time series were not accounted for, they were all significantly related to the lunar index (Figure 4). There was no significant positive relationship with the lunar index for species feeding on herbs or mosses (Table 2). Neither were they significantly negatively related to the solar index (Table 2).

**FIGURE 5:**
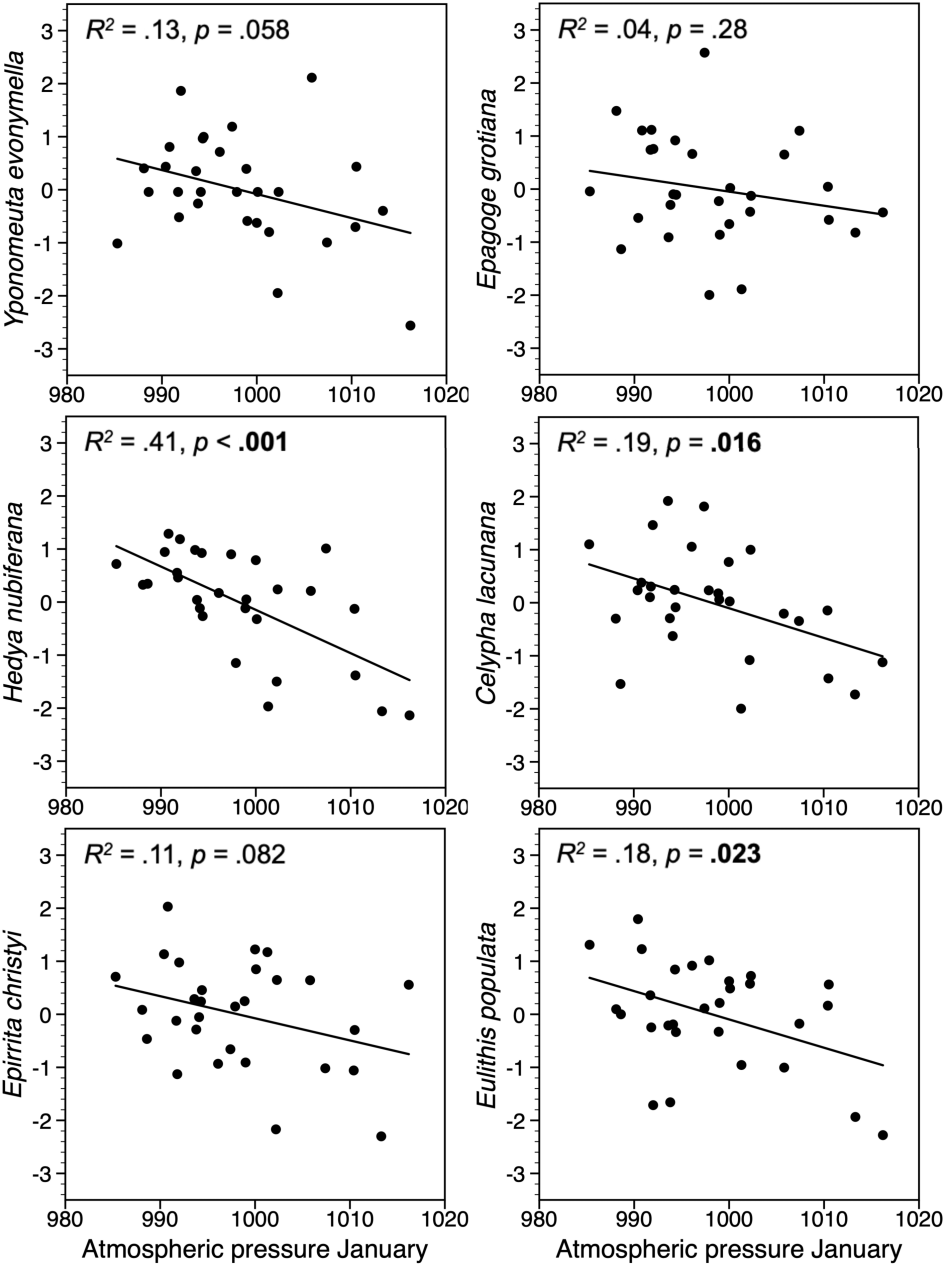
Standardized values of annual change (first difference) of the log‐transformed trapping indices of six moth species feeding on deciduous woody plants, plotted against mean January atmospheric pressure in the previous year. Significant *p*‐values are in bold.

**FIGURE 6:**
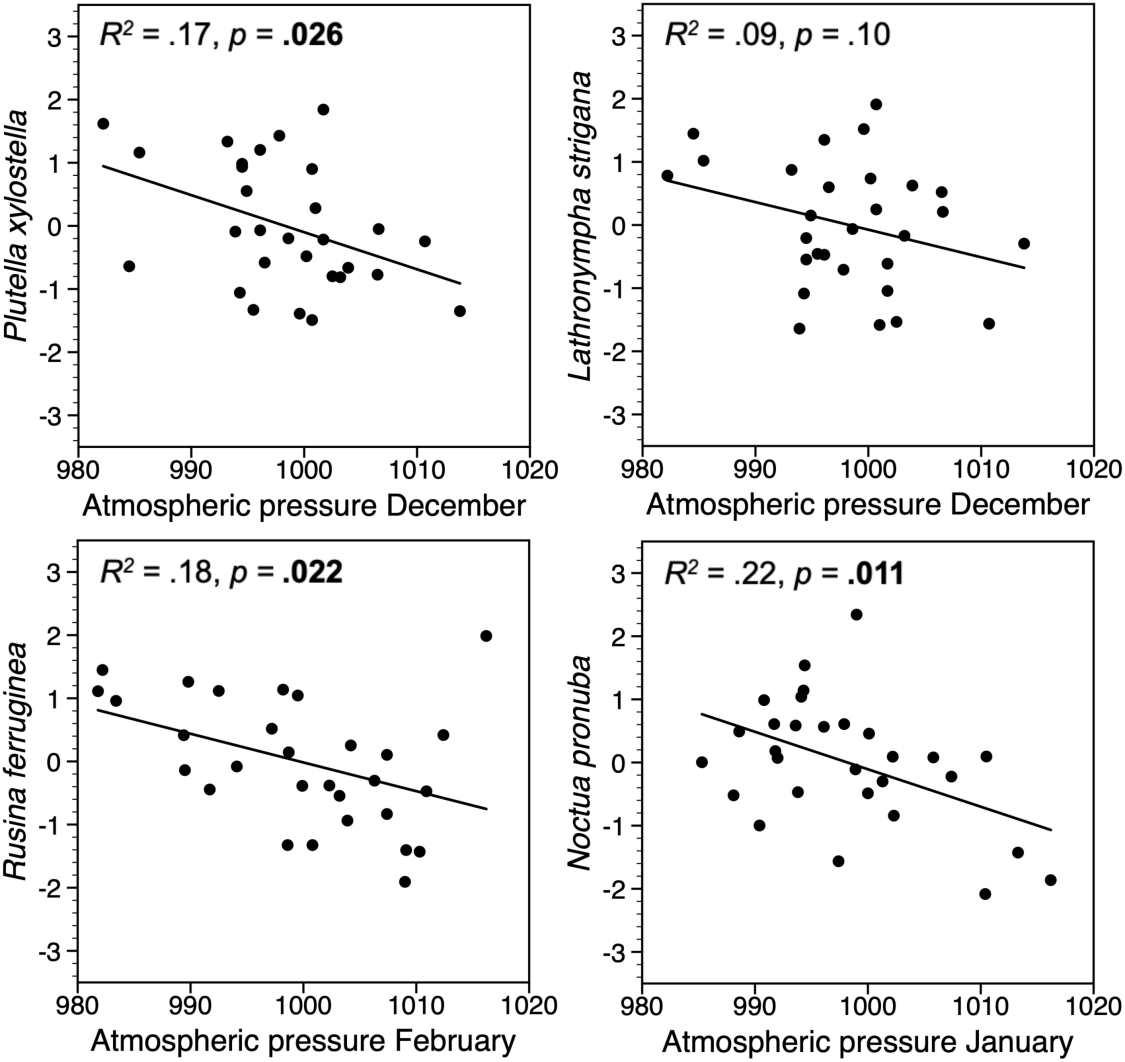
Standardized values of annual change (first difference) of the log‐transformed trapping indices of four moth species feeding on herbs, plotted against mean December (two upper panels), January (lower right panel), or February (lower left panel) atmospheric pressure in the previous year. Significant *p*‐values are in bold.

**FIGURE 7:**
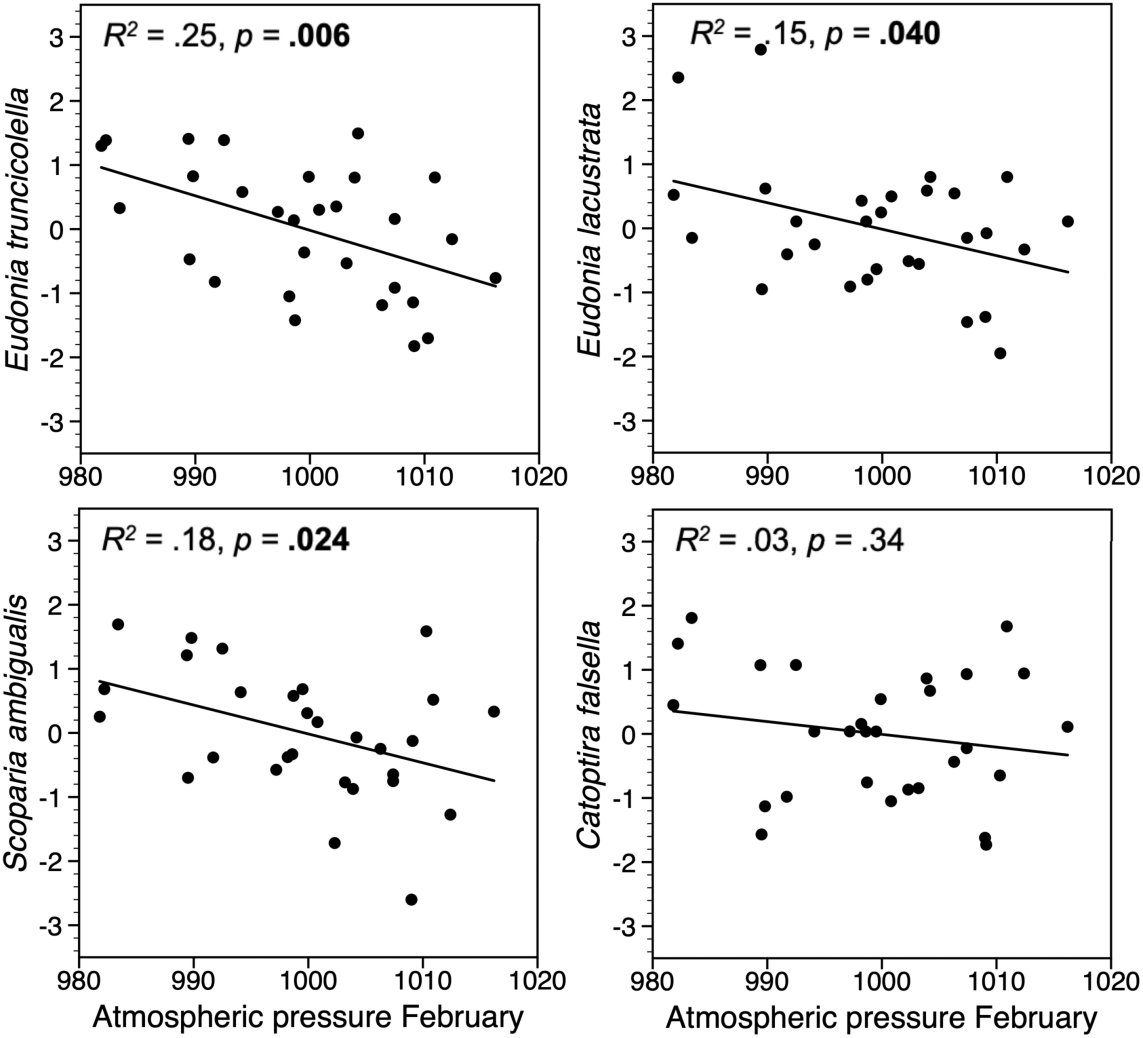
Standardized values of annual change (first difference) of the log‐transformed trapping indices of four moth species feeding on mosses, plotted against mean February atmospheric pressure in the previous year. Significant *p*‐values are in bold.

When using annual change (first difference) of the log‐transformed trapping indices, 9 of the 14 species showed a significant negative relationship with winter atmospheric pressure in the previous year (Figures 5, 6, 7), and there was a similar tendency for two other species (*p* < .1). For all species feeding on deciduous woody plants, the best correlation was with mean atmospheric pressure in January, but the relationship was significant only for three of them (Figure 5). Three of the four herb‐feeding species correlated negatively with atmospheric pressure, one in December, one in January, and one in February (Figure 6). For the species feeding on mosses, there were three that correlated significantly with atmospheric pressure in February (Figure 7).

In a linear mixed regression model with annual change of the log‐transformed trapping indices as response variable, there was a positive relationship with the lunar index and a negative relationship with January and February atmospheric pressure (Table 3). There also was a significant effect of the interaction between feeding guild and atmospheric pressure in December and February (Table 3), but not between feeding guild and the lunar index (*p* = .21 in the full model). The solar index did not contribute significantly, neither together with the lunar index (*p* = .32), nor as a substitute for this predictor (*p* = .97).

**TABLE 3:** Results from a mixed linear regression model with the annual change (first difference) of the log‐transformed trapping index for 14 common moth species in a 30‐year light‐trapping study in South Norway year *t* as response variable.

Species	Estimate	*SE*	df	*F*	*p*
Intercept	52.906	10.907	1		
Feeding guild			2	1.04	.386
Lunar index	0.081	0.034	1	5.65	**.018**
Pressure December	−0.001	0.008	1	0.01	.947
Pressure January	−0.031	0.007	1	17.40	**<.001**
Pressure February	−0.022	0.006	1	13.94	**<.001**
Guild × December			2	4.09	**.018**
Guild × January			2	2.58	.077
Guild × February			2	5.96	**.003**
Species (random)					**<.001**

*Note*: Explanatory variables are feeding guild (fixed effect, 3 levels), the lunar nodal phase index, mean atmospheric pressure in the winter months December (year *t* − 2), January (year *t* − 1), and February (year *t* − 1), and interactions between feeding guild and other predictors. Species was included as random effect. Only variables with *p* < .1 are included in the model. Significant *p*‐values are in bold.

## 4 DISCUSSION

In this study, we tested for relationships between indices of moth abundance and factors that affect surface fluxes of muons, the compound of secondary cosmic rays that most strongly affects life on Earth. The hypothesis was that ionization would increase the protein digestibility of food plants, in accordance with the plant stress hypothesis. Although our analyses were based on only one fixed sampling site each year, we found support for the predicted relationship with both the 9.3‐year lunar nodal phase cycle and atmospheric pressure in the previous winter. Three of six species that feed on deciduous woody plants were positively related to the lunar index, and for the remaining three, there was a marginally non‐significant relationship. The predicted negative correlation between moth indices and previous winter atmospheric pressure was supported for 9 of the 14 moth species. Because of the 1‐year time lag, the latter pattern is unlikely to be due to direct weather effects on the moths.

The lunar index explained 22%–24% of the variation in the population indices of 4 of the 6 months feeding on deciduous woody plants, and 33%–48% for the remaining two. We regard this as sufficient support for the hypothesis about a link between moth abundance and the lunar index. There was no time lag, in contrast to an analysis of a 120‐year time series based on autumnal moth defoliation reports from high‐altitude areas in Scandinavia, where the moth index lagged 3 years behind the lunar index (Selås, 2014). The difference between the two studies may reflect that population peaks last longer at high altitudes, where muon fluxes are higher, and plant recovery is likely to be hampered by low temperatures and short growing seasons.

We found no relationships between the lunar index and population indices of moth species feeding on herbs or mosses. However, the difference between feeding guilds was not sufficient to give significant interaction effect in the linear mixed model with annual change of the log‐transformed population indices as response variable. One reason could be that there were some asynchronous population fluctuations within each feeding guild. Temporal asynchrony has been reported for sympatric and cyclic populations of autumnal moth and winter moth *Operophtera brumata* feeding on mountain birch *Betula pubescens* in Fennoscandia (Tenow et al., 2007). Possible causes for such asynchrony could be interspecific differences in life‐history traits (Mjaaseth et al., 2005), dispersal (Vindstad et al., 2019), vulnerability to predation (Klemola et al., 2009), tolerance to unfavorable weather conditions (Bylund, 1999; Jepsen et al., 2008), or ability to cope with different chemical compounds in the food plants (Kaitaniemi et al., 1999).

Earlier attempts of linking approximately 10‐year moth cycles to the solar cycle (Selås et al., 2004) were rejected because the moth species in question tended to have shorter cycle period than 11 years (Myers & Cory, 2013; Nilssen et al., 2007). However, for the herbivorous porcupine *Erethizon dorsatum* in eastern Quebec, there was a clear signal for a cycle period of 11 years during a period of 130 years (Klvana et al., 2004). Furthermore, in the study of Selås (2014), the autumnal moth index was negatively related with the 11‐year solar index when the 9.3‐year lunar signal was controlled for. During our study period, there was no extra explanatory power of the solar index because of its strong correlation with the lunar index. Muon fluxes depend on both solar activity and the lunar nodal phase, but the relative strength of these two forces may vary geographically, depending on atmospheric protection and distance to the auroral oval (Selås, 2014, and references therein). In areas with better protection against cosmic rays, the 11‐year cycle in solar activity may be more important for surface muon fluxes than the lunar nodal phase cycle. This could be the reason why the porcupine in Quebec fluctuated with a periodicity of 11 years.

To our knowledge, our study is the first to report a negative correlation between herbivore performance and atmospheric pressure in the previous winter. During our study period, mean atmospheric pressure was lowest in January, followed by December. Accordingly, nine of the 10 species feeding on vascular plants correlated best with atmospheric pressure in one of these 2 months. In contrast, population indices of the moss‐feeding species were negatively correlated only with atmospheric pressure in February. A possible explanation is that mosses, which lack dormancy (Glime, 2021), are most sensitive to ionization in late winter, due to dehydration caused by increased photosynthesis. The moss‐feeding species all belonged to the family Crambidae, but we find it unlikely that the pattern of population fluctuation in nocturnal moths is a taxonomy‐related phenomenon. By analyzing a 26‐year time series on moth light‐trapping indices from Finland, Kozlov et al. (2010) concluded that host‐plant quality or quantity may regulate populations of some moth species, whereas taxonomy seemed to have minor impact.

There was a rather regular fluctuation in the population indices of several of the investigated moth species, not only for those feeding on deciduous woody plants. There was no consistent periodicity for species feeding on herbs, but for three of four species feeding on mosses, there was a cycle period of approximately 7 years. This can hardly be explained by lunar or solar cycles. Interestingly, a 7‐year cycle has been reported also for the moss‐feeding wood lemming *Myopus schisticolor* (Selås et al., 2013). Clough (1920) identified an approximate 7‐year cycle in winter atmospheric pressure in northern Europe. During our study period, there actually was a positive autocorrelation in February atmospheric pressure at lag 7 years, but not significant. It is possible, however, that atmospheric pressure is not a perfect inverse proxy of surface muon fluxes, which also depend on stratospheric temperatures (de Mendonca et al., 2016).

Observed population fluctuations of moths may not match the lunar nodal phase cycle or atmospheric pressure indices perfectly, because of the modulating effect of other factors, in particular weather. The amplitude of herbivore population fluctuations seems to be dampened in periods with high temperatures (Büntgen et al., 2020; Cornulier et al., 2013; Yan et al., 2013), a phenomenon that may also have affected our results (Burner et al., 2021). Anyway, cyclicity is most likely to be observed for phenomena related to the lunar or solar index, because fluctuations in atmospheric circulations and related weather patterns are less regular. Grouse, for instance, are known to exhibit population cycles with periods of both 7–8 and ca. 10 years (Moss & Watson, 2001), but the 7–8‐year cycles are sometimes referred to as quasi‐cycles (Haydon et al., 2002). In South Norway, a significant 3–4‐year fluctuation in atmospheric pressure in June has been hypothesized to affect flower bud induction in perennial plants grazed by small rodents with 3–4‐year population cycles (Selås, 2016). But here too, deviations from the cyclic pattern are common.

A study period of 30 years is too short to reveal exact cycle periods of herbivores with a decadal cycle pattern, but we find the reported mean period of 9.3 years in some other studies, in particular that of the larch budmoth (1145 years; Esper et al., 2007), so convincing that we think a possible link to the lunar index deserves further attention. Alternative hypotheses should always be welcome, but presently we do not have any other possible explanations for the 9.3‐year population cycles than the proposed impact of cosmic ray muons. We regard the negative relationship with winter atmospheric pressure in our study as further support for the cosmic ray hypothesis. Other factors, such as harsh weather events or predators and parasitoids, may contribute to modulate the fluctuation patterns, for example, by enhancing cycle amplitudes or delay some peaks, but they are unlikely to be the ultimate cause of relationships with the lunar nodal phase, solar activity, or atmospheric pressure.

It is noteworthy that cycle periods in moth populations appear to be related to geographical location or vegetation type, rather than to moth species (Johnson et al., 2006; Li et al., 2015). Our study was carried out at relative low elevation and also at some distance from the auroral oval. Here, a decadal fluctuation pattern is more likely to be disturbed by confounding factors than in areas with lower atmospheric and geomagnetic protection against cosmic ray muons. Our prediction then is that the lunar signal will be stronger in time series of moth populations at higher altitudes, in particular in alpine areas, and at higher geomagnetic latitudes, such as in the boreal forests of northern America. However, thorough investigations of the proposed physiological mechanisms are needed for the cosmic ray hypothesis to be widely accepted. We hope our contribution can encourage plant and animal physiologists to initiate such studies.

## AUTHOR CONTRIBUTIONS

**Vidar Selås:** Data curation (equal); formal analysis (lead); writing – original draft (lead); writing – review and editing (equal). **Sverre Kobro:** Data curation (equal); formal analysis (supporting); writing – original draft (supporting); writing – review and editing (equal).

## CONFLICT OF INTEREST

The authors declare no conflict of interest.

## Data Availability

The light‐trapping moth data are available on https://doi.org/10.5281/zenodo.5553031.
